# Congenital Zika syndrome and living conditions in the largest city of northeastern Brazil

**DOI:** 10.1186/s12889-022-13614-x

**Published:** 2022-06-20

**Authors:** Marcos Paulo Almeida Souza, Márcio Santos da Natividade, Guilherme Loureiro Werneck, Darci Neves dos Santos

**Affiliations:** 1 Instituto de Saúde Coletiva, Federal University of Bahia, Salvador, Bahia Brazil; 2 Department of Surgery, University Hospital of Lagarto, Federal University of Sergipe, Lagarto, Sergipe Brazil; 3 Instituto de Estudos em Saúde Coletiva, Federal University of Rio de Janeiro, Rio de Janeiro, Brazil; 4 Instituto de Medicina Social, State University of Rio de Janeiro, Rio de Janeiro, Brazil

**Keywords:** Congenital Zika syndrome, Ecological study, Social determinants of health

## Abstract

**Background:**

The Zika virus (ZIKV) epidemic hit Brazil in 2015 and resulted in a generation of children at risk of congenital Zika syndrome (CZS). The social vulnerability of certain segments of the population contributed to the disproportional occurrence of CZS in the Brazilian Northeast, the poorest region in the country. Living conditions are essential factors in understanding the social determination of CZS, which is embedded in a complex interaction between biological, environmental, and social factors. Salvador, the biggest city in the region, played a central role in the context of the epidemic and was a pioneer in reporting the ZIKV infection and registering a high number of cases of CZS. The aim of the study was identifying the incidence and spatial distribution pattern of children with CZS in the municipality of Salvador, according to living conditions.

**Methods:**

This is an ecological study that uses the reported cases of ZIKV and CZS registered in the epidemiological surveillance database of the Municipal Secretariat of Health of the city of Salvador between August of 2015 and July of 2016. The neighborhoods formed the analysis units and the thematic maps were built based on the reported cases. Associations between CZS and living conditions were assessed using the Kernel ratio and a spatial autoregressive linear regression model.

**Results:**

Seven hundred twenty-six live births were reported, of which 236 (32.5%) were confirmed for CZS. Despite the reports of ZIKV infection being widely distributed, the cases of CZS were concentrated in poor areas of the city. A positive spatial association was observed between living in places with poorer living conditions and births of children with CZS.

**Conclusions:**

This study shows the role of living conditions in the occurrence of births of children with CZS and indicates the need for approaches that recognize the part played by social inequalities in determining CZS and in caring for the children affected.

## Background

Zika virus (ZIKV) infection and its effects on infant development emerged around 5 years ago as a new and serious public health problem in Brazil and the world [1, 2]. Epidemiological and clinical evidence have suggested an association between ZIKV infection during pregnancy and neurological alterations in newborns [3–5]. This causality relationship has achieved a consensus in the scientific literature, refuting other hypotheses about vaccines and larvicides [6–9].

In the Brazilian epidemic, microcephaly in newborns was identified as the first consequence associated with intrauterine infection by ZIKV [10]. The 28-fold increase in births with microcephaly in relation to the mean of previous years contributed to the Ministry of Health declaring a nationwide public health emergency in November of 2015 [11, 12]. Given the seriousness of the situation and the occurrence of cases in other countries and potential global spread, in February of 2016 the World Health Organization (WHO) declared that the situation in Brazil was a public health emergency of international interest [13]. Subsequently, multiple congenital alterations associated with ZIKV infection were observed, defining the condition of congenital Zika syndrome (CZS). Although its spectrum is not yet fully known, the following characteristics have already been described: visual alterations, craniofacial disproportion, limb contractures, and cerebral calcifications and other lesions, including in the absence of microcephaly [14, 15].

Most of the cases of CZS reported between 2015 and 2016 were concentrated in the Brazilian Northeast (65.7%). The estimated prevalence ratio in the region was 12.6 confirmed cases per 10 thousand live births in 2015 and 7.1 in 2016, while at a national level the respective estimates were 3.8 and 3.1 per 10 thousand live births [16]. High ZIKV infection rates also occurred in the Central-West and Southeast regions of the country [17]. However, they did not translate to the same extent into births marked by CZS, demonstrating an asymmetry in the population distribution of the syndrome and suggesting a complex interaction between biological, environmental and social factors [18, 19].

Despite the socioeconomic advances over the last decade, the Northeast region still has the lowest human development index (HDI), reflecting poorer living conditions [20]. Given the causality relationship between intrauterine ZIKV infection and CZS, it is worth considering the social determinants of the dynamic of mosquito-borne diseases [21, 22]. Despite the lack of consensus in the current literature about the association between poverty and the multiple mosquito-borne diseases [23], the ZIKV epidemic in Brazil revealed this relationship in the sense that its consequences predominantly affected the poor, black population living on the outskirts of the cities [24].

These affected population groups were exposed to the mosquito vector for decades, considering that they resided in areas with household overcrowding and a lack of adequate sanitary and social infrastructure [24]. The presence of a favorable tropical climate combined with the lack of basic services such as the connection to a water network system with constant supply, regular garbage collection, sewage network, and effective rainwater drainage systems promotes stagnant water constituting the ideal habitat for reproduction of the mosquito vector. These families live on the fringes of public policies, which increases the risk of ZIKV infection and its consequences [25].

Among the states of the Brazilian Northeast, Pernambuco was the epicenter of the epidemic, with the highest number of cases reported between 2015 and 2016 [26]. The cases of microcephaly in Recife, the state capital, were predominantly located in areas with the lowest income and poorest living conditions [27]. The state of Bahia recorded the second highest number of confirmed cases [28], concentrated in the capital Salvador [29].

In the study of Souza et al. [27], only the occurrence of microcephaly, without considering CZS, was related with the urban socioeconomic conditions in Recife, where income was used as the only indicator of living conditions. Despite its relevance, income is an insufficient indicator for representing the complexity of the relationships of the social determinants implied in the occurrence of CZS.

In light of that gap, this study includes new factors to examine the effect of living conditions over the main consequence of ZIKV infection. Therefore, it aimed to identify the incidence and spatial distribution pattern of children with CZS in the municipality of Salvador, according to living conditions.

## Methods

### Design, database, and population of the study

This is a cross-sectional ecological population-based study that used secondary data from the surveillance system of the Municipal Secretariat of Health of Salvador. The study population is composed of children born between August 1st of 2015 and July 31st of 2016 reported as having microcephaly and CZS. This involved the database of the Record of Public Health Events developed by the Ministry of Health of Brazil, based on the surveillance protocol during the public health emergency, in which all health services, public and private, were responsible for reporting new cases of microcephaly and other congenital anomalies [30].

### Case definition

The current definition of a suspected case of CZS employed by the Brazilian Ministry of Health considers anthropometric or clinical criteria and image exams [12]. Etiological confirmation depends on positive laboratory results for ZIKV, which were predominantly unavailable at the time of the outbreak. Although cephalic perimeter (CP) was immediately adopted as the primary criterion for screening cases suspected of congenital abnormalities by ZIKV between 2015 and 2016, CZS occurs independently of the presence of microcephaly [14, 31]. Indeed, the CP criterion was modified three times during the outbreak [16, 17]. Therefore, to avoid multiple criteria, this study only classified the children using the results of perinatal and post-natal neuroimaging exams, independent of the presence of microcephaly.

The inclusion criterion adopted for the study population was having been born at maternity clinics in Salvador and having a residential address in the same municipality. It was assumed that mothers of children born in Salvador lived in this city during their pregnancy. The subjects were classified according to findings of intracranial calcifications, ventriculomegaly, dysgenesis or agenesis of the corpus callosum, lissencephaly, and an increase in periventricular echogenicity and in the quantity of cerebrospinal liquid in the brain [32] compatible with CZS revealed by imaging exam.

An individual analysis of the imaging exams recorded in the database identified three categories of participants in relation to CZS:

- **Confirmed** – presence of alterations suggestive of CZS;
- **CZS discarded –** normality in the imaging exams or alterations not suggestive of CZS;
- **Incomplete investigation (suspected cases) –** no result for the imaging exam or the aforementioned exam was not conducted.

### Study scenario

**Fig. 1:**
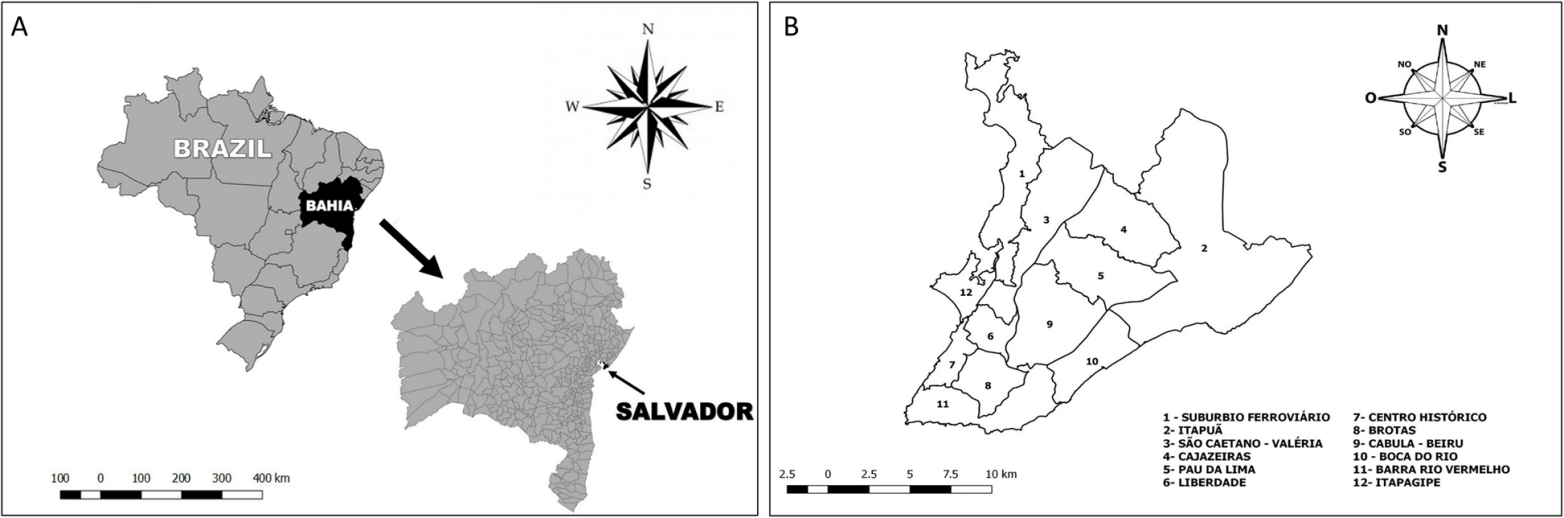
**A** – Location of the biggest city in the Northeast – Salvador, State of Bahia, Brazil. **B** – Health Districts of the city of Salvador

The estimated population of 2.8 million inhabitants in 2018 in the urban area of Salvador places it as the fourth biggest city in Brazil (Fig. 1). The territory covers 692,818 km^2^, with a population density of 3859.44 inhab./km^2^ [33]. The HDI is 0.759 and only 38% of the population is covered by primary healthcare services [34]. Salvador has 12 health districts (HDs), an administrative-operational unit of the public health system, and 160 neighborhoods belonging to these HDs, which were used as spatial analysis units.

### Living conditions index

Based on the 2010 Demographic Census, carried out through the SIRGAS 2000 system of the Brazilian Institute of Geography and Statistics (IBGE) [35], the neighborhoods of Salvador were classified according to the living conditions of the respective populations based on the aggregation of five indicators to form the living conditions index (LCI). To build that index, the methodology of Paim et al. [36] was adopted, which employed five indicators as proxy variables for living conditions, based on data from the 1991 Demographic Census: **income, education, sanitation,** ***favela*,** and **inhabitants per room*.*** The **income** calculation considered the proportion of heads of household with a mean monthly income ≤ two minimum wages. **Education** considered the proportion of literate people aged between 10 and 14. **Sanitation** considered the percentage of homes connected to the general water supply. ***Favela*** considered the percentage of homes in a subnormal cluster (*favela*). **Inhabitants per room** considered the mean number of inhabitants per residence in relation to the mean number of rooms used as bedrooms [36].

In this study adaptations were needed to operationalize these last two indicators to build the LCI with the data available in the 2010 Demographic Census. Thus, for the ***Favela*** indicator the calculation occurred based on the “sector type” variable, where in that 2010 census code 1 represented the “census sector (CS) of the special subnormal cluster type.” For each neighborhood, the total CSs defined by code 1 constituted the numerator of that indicator while the denominator was represented by the total residences.

In relation to the **inhabitants per room** indicator, the “**mean number of rooms per residence”** and **“mean number of bedrooms per residence”** variables were not present in the 2010 census and were substituted by the **“inhabitants in permanent private residences”** and **“permanent private residences”** variables, with which it was possible to calculate the **“number of people per residence”** variable.

Continuing with the LCI calculation, the **inhabitants per room**, ***favela***, and **income** indicators of each neighborhood were distributed in ascending order of their values (the higher, the worse), while the **sanitation** and **education** indicators were arranged in descending order (the higher, the better). Next, each one received a score starting with the number 1, depending on the position occupied considering the ascending or descending order for building the LCI (Table 1).

**Table 1:** Construction of the Living Conditions Index

Indicator	Calculator	Interpretation	Arrangement
**Income**	Heads of household with income ≤2 minimum wages	The higher, the worse	Ascending order
**Favela**	% of houses in a subnormal cluster (*favela*) in relation to total residences		
**Inhabitant / Room**	mean n. of inhabitants per residence in relation to the mean n. of bedrooms per residence		
**Education**	Proportion of literate people from 10 to 14	The higher, the better	Descending order
**Sanitation**	% of residences connected to the general water supply		

Finally, the sum of the score of these five indicators resulted in a score (LCI score) for each neighborhood. Higher LCI scores correspond to the poorest living conditions. These scores were also organized in ascending order and grouped according to quartiles of relatively homogeneous neighborhoods, corresponding to population strata classified as high (1), intermediate (2), poor (3), and very poor (4) living conditions.

### Statistical analysis

The incidence of confirmed cases of CZS was calculated for every 10,000 live births, according to the neighborhood of residence. The number of live births per neighborhood in Salvador in the period studied was obtained from the Live Births Information System (SINASC).

Cases of CZS (reported and confirmed) were georeferenced using the QGIS software (QGIS Geographic Information System. Open Source Geospatial Foundation. https://qgis.org/en/site/) through the application programming interfaces (APIs) of Google Maps, a tool that transforms the text addresses stored in a database into geographical coordinates, in the form of latitude and longitude. These were spatially distributed on the cartographic map of the neighborhoods of Salvador in a shapefile format, obtained from the Urban Development Company of the State of Bahia.

Based on the specific geographical distributions of the cases, the Kernel ratio technique [37, 38] was applied, and then the thematic maps were built for the period studied. Simulations were run to test bandwidth, considering 800 m, 900 m, and 1000 m, where the 900 m distance was the one that presented the best image for visualizing the spatial distribution of the problem studied.

To examine the relationship between living conditions and CZS, the cases were aggregated by neighborhood. The incidence of CZS was calculated for the neighborhoods of the municipality of Salvador, dividing the sum of the number of confirmed cases of CZS from the corresponding period by the total number of live births from the same period, and multiplying the values by 10,000. With the aim of minimizing the instability of the gross rates resulting from small numbers of observations, the smoothing method was applied using the local empirical Bayes estimator [39, 40].

To proceed with the spatial analysis, a neighborhood matrix or adjacency weight matrix (close neighbors with at least one boundary point in common) was built, using the GeoDa 1.8 program. The existence of an association between the smoothed CZS rate and socioeconomic variables of the neighborhoods was assessed by applying spatial autoregressive (SAR) linear regression models. Given the presence of a spatial autocorrelation in the smoothed CZS rates, the modeling was adjusted by demographic density and incidence of ZIKV infection.

Categorical data were compared using Fisher exact test (2-sided) to test for differences between confirmed, discarded, and incomplete-investigation CZS groups. All the statistical analyses were conducted using version 16 of the Stata software (College Station, Texas, USA) and GeoDa 1.8, accepting a 5% significance level.

## Results

Between August 1st of 2015 and July 31st of 2016, 726 live births with suspected microcephaly were reported to the MSH of Salvador, Bahia. Of these, 490 reports (67.5%) presented results of some type of imaging exam (ultrasound, computed tomography, or magnetic resonance). Among these, 236 (48.2%) showed alterations in the imaging exams compatible with CZS, while 251 (51.8%) children exhibited results that were normal or not suggestive of CZS, this being the group classified as discarded. The absence of imaging exams or of any other information occurred in 236 (32.5%) reports, constituting the group classified as incomplete investigation (Table 2).

**Table 2:** Perinatal characteristics of the reports of the children and mothers, according to the definition of groups associated with the occurrence of CZS in Salvador, BA, between 08/01/2015 and 07/31/2016

Perinatal characteristics	Congenital Zika Syndrome (CZS)						***P*** value
	Confirmed (***n*** = 236)		Discarded† (***n*** = 254)		Incomplete investigation* (n = 236)		
	n	%	n	%	n	%	
**Children**							
**Sex (*n*** **= 723)**							
Female	134	56.8	162	63.8	138	59.2	†0.117
Male	102	43.2	92	36.2	95	40.8	*0.640
**Weight at birth (*n*** **= 696)**							
≥ 2500 g	143	61.6	169	68.1	154	71.3	†0.151
< 2500 g	89	38.4	79	31.9	62	28.7	*0.036
**Deaths**	10	4.2	0	0.00	13	5.5	†0.001
							*0.670
**Mother**							
**Race/Color (*n*** **= 598)**							
White	13	6.2	9	3.9	11	6.9	†0.281
Non-white	195	93.8	222	96.1	148	93.1	*0.833
**Gestational age (*n*** **= 663)**							
< 37 Weeks	75	33.3	13	5.6	32	15.6	† < 0.001
37–42 Weeks	150	66.7	220	94.4	173	84.4	* < 0.001
**STORCH (*n*** **= 598)**							
Positive	24	11.7	21	9.0	17	10.6	†0.431
Negative	182	88.3	211	91.0	143	89.4	*0.868
**Total**	236	32.5	254	35.0	236	32.5	

^†^CZS confirmed x Discarded

^*^CZS confirmed x incomplete investigation

In general, the female gender prevailed in our sample globally (60.0%) and among the three categories of participants (CZS confirmed, discarded and incomplete investigation). The frequency of prematurity was 33.3% among the children with CZS, approximately six times higher than those without CZS (5.6% - *p* < 0.001) and 2.2 times higher than the percentage observed among the group with an incomplete investigation (15.6% - *p* < 0.001). A low birth weight also predominated among children affected by CZS (38.4% - *p* = 0.036/suspect cases). The presence of infections in pregnancy (STORCH – syphilis, toxoplasmosis, others [HIV, B19 parvovirus], rubella, cytomegalovirus, and herpes virus) was verified in 11.7% of the mothers of children with CZS. A consistent majority of non-white mothers was observed in the three groups investigated: 93.8% among children affected with CZS, 96.1% among those free of the syndrome, and 93.1% in the group with an incomplete investigation (Table 2).

Twenty-three deaths were recorded in the period, of which 10 (4.2%) were among children with CZS (*p* < 0.001/CZS discarded) and 13 (5.5%) were in the group without a complete investigation. There was no record in those who were free of CZS (Table 2). Greater variability and a lower median for CP were observed among children with CZS (30.0 cm) in relation to those discarded for CZS and those not investigated using an imaging exam, both of which had a median of 32.0 cm.

**Fig. 2:**
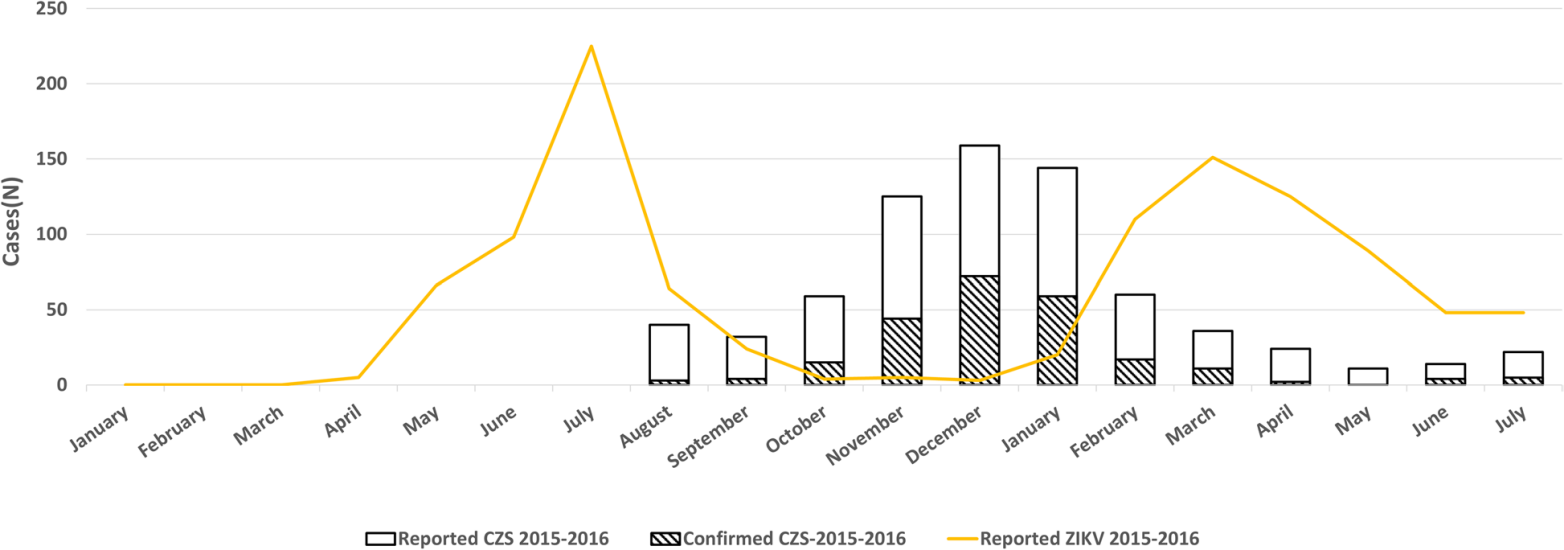
Distribution of suspected cases of ZIKV infection and reported and confirmed cases of CZS between August 1st of 2015 and July 31st of 2016, by month of occurrence, in the municipality of Salvador, Bahia, Brazil

In relation to the reports of ZIKV infection, 494 suspected cases were observed in 2015, when the reports began in April (1%) and the peak was in July (45.1%). By July of 2016, there were a total of 591 reports, with March being the month with the highest frequency (25%) of reports in that year. For the suspected occurrence of CZS, the reports began in August of 2015 (9.6%), reaching the highest frequency in December (38.3%). The same pattern was observed for the confirmed cases of CZS. In December of 2015, 45% of all reported cases were confirmed to have CZS, the highest proportion in the analysis period. From January of 2016, there was a reduction in reports of CZS, and despite a new peak of suspected ZIKV infections, this situation did not translate into an increase in the number of cases of CZS. The increase in cases was considered as a second wave of ZIKV infections in March of 2016 (Fig. 2).

**Fig. 3:**
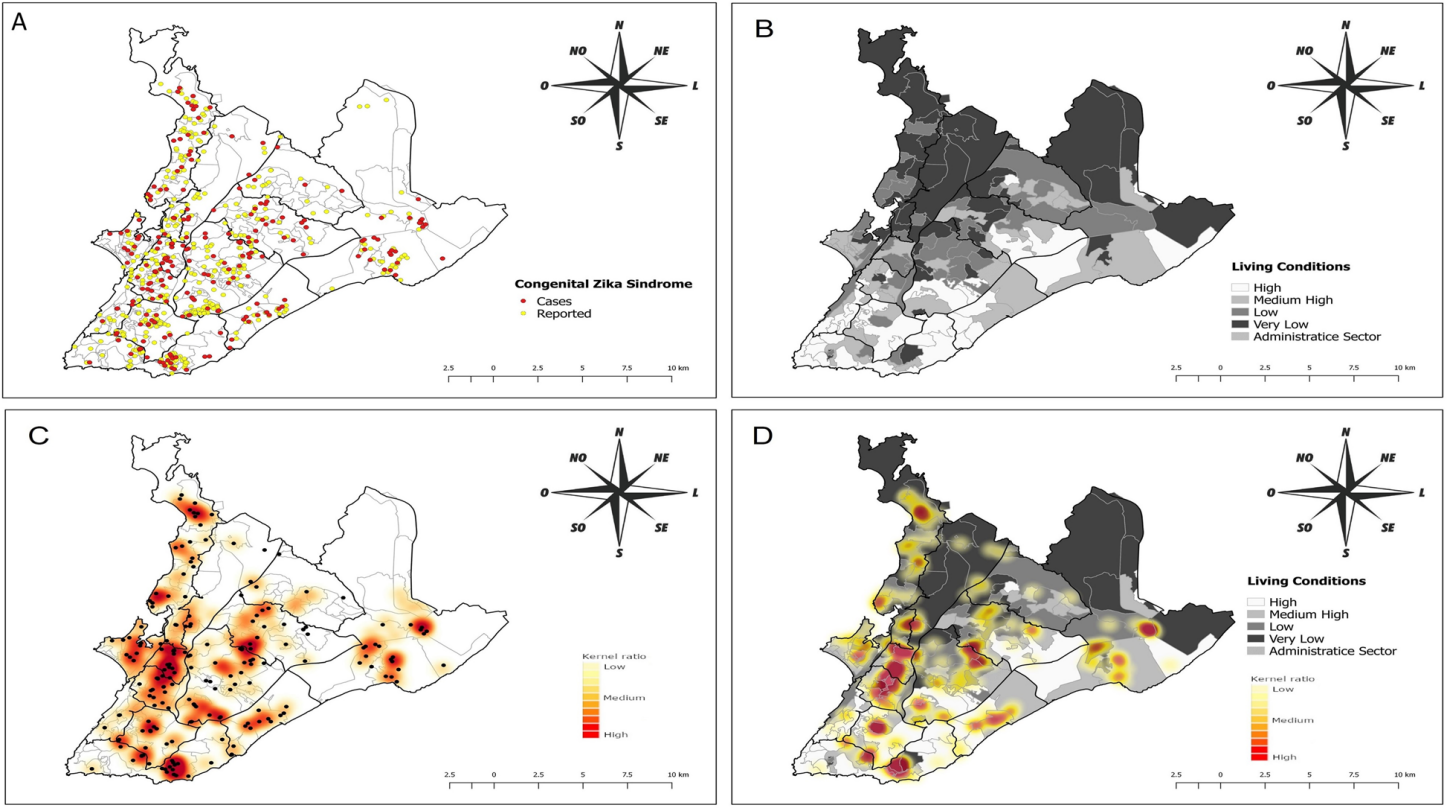
Spatial distribution of suspected and confirmed cases of CZS according to living conditions in the municipality of Salvador, Bahia, Brazil between August 1st of 2015 and July 31st of 2016. **A** - Reported and confirmed cases of CZS; **B** – Strata of living conditions in the city of Salvador; **B** - Kernel ratio for confirmed cases of CZS; **D** - Kernel density for confirmed cases of CZS according to living conditions

The spatial distribution of children born with suspected and confirmed CZS in the municipality of Salvador primarily occurred in the west and southwest regions (Fig. 3A). The distribution of the living conditions strata is presented in Fig. 3B. The analysis of the Kernel ratio based on the distribution of confirmed cases of CZS showed extensive areas of risk, especially located in the HDs of São Caetano, Subúrbio Ferroviário, Barra Rio Vermelho, Cabula, and Itapuã (Fig. 3C). It is observed that the biggest risk zones for CZS, when superimposed on the living conditions index, are present in the places with poorer socioeconomic conditions, even constituting islands of poverty, that is, areas of very poor living conditions surrounded by strata of medium-high and high living conditions (Fig. 3D).

The confirmed cases of CZS in the areas with very poor living conditions represented 26.5% of the total. When added to the cases in the places with poor living conditions, these represent more than half of the cases (55.5%). Only 12.8% of the children with CZS were in areas considered as having high living conditions, a difference of 4.3 times in relation to the sum of the areas that translate precarious living conditions. In the linear regression, the association between the LCI and the incidence of confirmed cases of CZS was expressive (β = 0.75; *p* < 0.006), controlled by the incidence of suspected ZIKV infection, an indicator of the level of basal exposure to *Aedes aegypti*. This means that for every one unit increase in the LCI there is a 0.75 rise in the incidence of CZS per 10,000 live births (Table 3).

**Table 3:** Spatial autoregressive (SAR) linear regression model between the living conditions index and the incidence of CZS in the neighborhoods of Salvador-BA, according to the incidence of suspected ZIKV infection and demographic density

Variables	Bivariate SEM model	
	β	***p*** value
Living conditions index (score)	0.75	0.0058
Incidence of suspected ZIKV infection	5.63	0.0029
Demographic density	0.54	0.0000

## Discussion

We observed a positive spatial association between living in places with poorer living conditions and live births with CZS. The cases of CZS were concentrated in the most impoverished areas of the city of Salvador. This spatial distribution pattern of the incidence of CZS due to maternal ZIKV infection reveals the contribution of structural aspects of social vulnerability to the occurrence of this phenomenon [9, 24, 41].

At the start of the epidemic in 2015, Paploski et al. [42] demonstrated a space-time association between the emergence of an acute exanthematous disease in Salvador and the birth of children with microcephaly. It was suggested that this exanthematous disease was caused by ZIKV, resulting in a higher number of newborns with microcephaly after 30–33 weeks of reporting ZIKV infection. The nationwide universal surveillance only started in February of 2016. However, the State of Bahia was the first to adopt obligatory reporting of ZIKV, advancing beyond the concept of acute exanthematous disease and differentiating it from other arboviruses such as dengue and chikungunya [43]. This surveillance placed Bahia, as the place in Brazil where ZIKV was discovered [44], and its capital, at the forefront of the fight against the epidemic and its consequences.

Our findings reveal that the temporal distribution of the infection and of CZS in Salvador followed the same pattern as the entire Northeast region [45], with a peak occurrence of CZS in December of 2015. Moreover, our data demonstrate an increase in cases of ZIKV infection between January and March of 2016, but this did not correspond to children being born with CZS.

It has not yet been fully elucidated why cases of CZS were concentrated in the Northeast region during the first wave of infection, while the second wave of infection did not translate into a proportional increase in cases of CZS both in the Northeast and in other regions of Brazil [45, 46]. However, the cumulative evidence suggests a multifactor interaction between biological conditions (the number of people susceptible to ZIKV and vector mutations), environmental conditions (favorable climate and hot-humid seasons), and socioeconomic conditions (high demographic density, precarious housing conditions, and poverty) [18, 41].

Although 11.7% of confirmed CZS showed positive results for STORCH infections, we chose to keep them in this group because most ZIKV infection are asymptomatic [47], at the time peak of the epidemic laboratory testing was predominantly unavailable, had cross-reactions with other flaviviruses, financial and logistical limitations for large-scale use [48], in addition to the possibility of co-infection which may imply the potential of enhancement congenital abnormalities in ZIKV infections [49] and underreporting that occurred due to co-circulation with other arboviruses [50].

Approximately 55% of cases of CZS in Salvador occurred in regions with precarious living conditions, a rate 4.3 times higher compared with areas with high living conditions, resulting in a direct association between poorer socioeconomic conditions and the incidence of CZS. This same relationship was found in Recife, in Pernambuco, which was heavily affected by the epidemic of newborns with microcephaly [27]. However, the difference in Recife was greater, with only 2% of confirmed cases of CZS being located in the richest regions of the city. The literature suggests that awareness, availability, and access to health services, as well as contraception methods, delayed pregnancy, and interrupted pregnancy by women with better socioeconomic conditions, would probably explain the low percentage of CZS in this privileged segment [51, 52].

During the epidemic, a 108% increase in requests for abortifacients was recorded [53], however the National Health Surveillance Agency confiscated some of those drugs due to the illegality of the practice in the country [54]. In Brazil, abortion is only permitted to save the mother’s life or in cases of rape or anencephaly [55]. Despite the reduction in hospitalizations due to abortion complications in the public health administrative records, such data do not cover safe abortions, which present a lower number of complications, suggesting that there may have been a selection of women from a higher social class in the decision to interrupt pregnancy [52].

Moreover, other structural factors that are asymmetrically distributed among population groups contribute to the increase in the risk of ZIKV infection and its consequences, with an accentuated economic and social impact for families affected by CZS [56, 57]. In our study, 93.8% of the mothers of children with CZS were classified as non-white and in the Northeast region this percentage corresponded to 83% of the population [45]. It warrants mentioning that in 2016, 85% of the female population living in Salvador stated that they were non-white [58], making it the Brazilian state capital with the highest percentage of non-white women.

When analyzing the socioenvironmental vulnerability of white and black pregnant women in Salvador in the period of the ZIKV epidemic, Santana et al. [59] observed that 31.6 and 34.5% of black women lived in areas with poor and very poor living conditions, respectively. In contrast, the white pregnant women predominantly lived in places with high (35.3%) and intermediate (29.4%) living conditions. Thus, the study suggests that white skin is predominantly associated with better education, income, and housing indicators and might be a protective factor for a child being born with CZS.

Nationwide, more non-white than white individuals live on the outskirts of the cities with a high household density, they have a 50% lower income, and have twice as much chance of living in houses with no garbage collection or sewage system. A greater proportion of non-whites are also observed living in places with no connection to the general water supply [60]. In Recife, sewage system (2.2x), garbage collection (1.96x), and houses in poor areas of the city (1.89x) were associated with a greater relative risk of microcephaly associated with ZIKV [61].

This study presents some limitations. For that reason, the data presented here should be interpreted with caution. We recognize that the municipality of Salvador presents its own particularities regarding the way it is architecturally and urbanistically organized, which restricts the extrapolation of these results. Worth highlighting is the dependency on the quality and availability of data from the health surveillance systems. Moreover, only reports of suspected ZIKV infection were considered. This is a new disease, in which only 20% of people infected develop symptoms, which may be confused with those of other arboviruses such as dengue fever and chikungunya. At the time there was a lack of laboratory exams for ZIKV due to the high cost of the laboratory kits and inconsistent results because of cross reactions with other arboviruses during the epidemic.

The definition of confirmed cases of CZS was based solely on neuroimaging exams, which despite not being specific, were frequently observed in this syndrome [15]. Such neurological alterations in the period of the epidemic were used as a proxy for CZS. Approximately one-third of the suspected cases in the study were not investigated. We can presume that at least some of these cases are of children with CZS, which may have led to underestimation in our analyses.

Finally, this is an ecological study of spatial aggregation subject to the effects of scale (aggregation of areas), imprecise definition of its boundaries, incomplete or non-registered addresses, and the possibility of some intra-area heterogeneity due to the use of pre-defined geographical-administrative divisions as an analysis unit (neighborhoods), and not more homogeneous areas in terms of socioeconomic conditions. This last restriction is particularly important due to the presence of *favelas* and middle/upper class neighborhoods in the same region.

## Conclusions

Our study shows the relationship between precarious living conditions and the incidence of CZS in the city of Salvador. As of 2016, the incidence of ZIKV infection and CZS were greatly reduced by the increase in immunity among the population [62]. However, although a new epidemic of similar proportions in unlikely, it is necessary to change the factors that increase the vulnerability of the poorest population living on the outskirts of the cities and thus improve the way of combatting *Aedes aegypti*. ZIKV infection can be considered a neglected tropical disease [63], and CZS, like any disease affecting child development, need effective actions of policymakers and the society to interrupt the deep socioeconomic inequalities and the disparities in access to health services to break the cycle of poverty and social exclusion experienced by people with disabilities and their families.

Besides environmental actions, identifying places where children with CZS live and risk maps would help the public authorities to outline strategies focused on monitoring this population group affected by the main consequences of the ZIKV epidemic. Thus, the infant growth and development surveillance system needs to be strengthened in the public health services, considering all children born throughout the epidemic and using prospective cohort studies to accompany the spectrum of the development of that generation of children. Integration between the health, education, and social assistance services is needed to provide social support and adequate inclusion into the school environment, which are essential for infant development.

However, implementing such changes is a challenge for Brazil, especially during the current pandemic caused by the new coronavirus (SARS-COV-2) and due to the budgetary restrictions following the adoption of fiscal austerity measures such as the freeze on public spending up to 2036 [64, 65].

## Data Availability

The data that support the findings of this study are available from the Center for Information and Epidemiological Surveillance of Salvador, but restrictions apply to the availability of these data, which were used under license for the current study, and so are not publicly available. Data are however available from the authors upon reasonable request and with permission of the staff of the Center for Information and Epidemiological Surveillance of Salvador (email: marcospaulo011@hotmail.com).

## Abbreviations

ZIKV: Zika virus
CZS: Congenital Zika syndrome
WHO: World Health Organization
HDI: Human development index
CP: Cephalic perimeter
HD: Health district
IBGE: Brazilian Institute of Geography and Statistics
LCI: Living conditions index
CS: Census sector
SINASC: Live Births Information System
API: Application programming interface
SAR: Spatial autoregressive linear regression
SARS-COV-2: New coronavirus
